# Genesis and Mechanism of Some Cancer Types and an Overview on the Role of Diet and Nutrition in Cancer Prevention

**DOI:** 10.3390/molecules27061794

**Published:** 2022-03-09

**Authors:** Nurkhalida Kamal, Muna Abdulsalam Ilowefah, Ayah Rebhi Hilles, Nurul Adlina Anua, Tahani Awin, Hussah Abdullah Alshwyeh, Sahar Khamees Aldosary, Najla Gooda Sahib Jambocus, Areej A. Alosaimi, Azizur Rahman, Syed Mahmood, Ahmed Mediani

**Affiliations:** 1 Institute of Systems Biology, Universiti Kebangsaan Malaysia (UKM), Bangi 43600, Malaysia; nurkhalida.kamal@ukm.edu.my (N.K.); nuruladlina720@gmail.com (N.A.A.); 2 Department of Food Technology, Faculty of Engineering and Technology, Sabha University, Sabha 00218, Libya; mona.milad2005@gmail.com; 3 Institute for Halal Research and Training (INHART), International Islamic University Malaysia, Kuala Lumpur 53100, Malaysia; ayah.hilles90@gmail.com; 4 Department of Chemistry, Faculty of Science, University of Benghazi, Qar Yunis, Benghazi 5341, Libya; awin_org@yahoo.com; 5 Department of Biology, College of Science, Imam Abdulrahman Bin Faisal University, Dammam 34212, Saudi Arabia; haalshuyeh@iau.edu.sa (H.A.A.); skdosary@iau.edu.sa (S.K.A.); aalosimi@iau.edu.sa (A.A.A.); 6 Basic & Applied Scientific Research Centre, Imam Abdulrahman Bin Faisal University, Dammam 31441, Saudi Arabia; 7 Ministry of Education, Tertiary Education, Science and Technology, MITD House, Phoenix 73544, Mauritius; najla.goodasahib@gmail.com; 8 Department of Clinical Pharmacy, Faculty of Pharmaceutical Sciences, UCSI University, Kuala Lumpur 56000, Malaysia; aziz@ucsiuniversity.edu.my; 9 Department of Pharmaceutical Technology, Faculty of Pharmacy, Universiti Malaya, Kuala Lumpur 50603, Malaysia

**Keywords:** cancer, diagnosis, diet, nutrition, phytochemicals, therapeutics

## Abstract

Cancer is a major disease with a high mortality rate worldwide. In many countries, cancer is considered to be the second most common cause of death after cardiovascular disease. The clinical management of cancer continues to be a challenge as conventional treatments, such as chemotherapy and radiation therapy, have limitations due to their toxicity profiles. Unhealthy lifestyle and poor dietary habits are the key risk factors for cancer; having a healthy diet and lifestyle may minimize the risk. Epidemiological studies have shown that a high fruit and vegetable intake in our regular diet can effectively reduce the risk of developing certain types of cancers due to the high contents of antioxidants and phytochemicals. In vitro and in vivo studies have shown that phytochemicals exert significant anticancer effects due to their free radical scavenging capacity potential. There has been extensive research on the protective effects of phytochemicals in different types of cancers. This review attempts to give an overview of the etiology of different types of cancers and assesses the role of phytonutrients in the prevention of cancers, which makes the present review distinct from the others available.

## 1. Introduction

Cancer is a disease which involves abnormal cell growth, with the potential to invade and metastasize other parts of the body. It has become a leading cause of death globally, causing nearly 10 million deaths in 2020. In 2018, approximately 9.6 million people died due to cancer [1]. As the prevalence of cancer continues to grow worldwide, new strategies are being sought for disease management. Cancer is a multifactorial disease and various factors such as diet and lifestyle, exposure to radiation, and hormonal factors can contribute to the development of this fatal disease [2]. Lifestyle factors, such as smoking, alcohol consumption, and dietary habits are considered to be significant contributing factors in the etiology of cancer and are also the main targets for primary prevention. The possible association between diet and development of cancer cannot be overlooked. For example, diets rich in red and processed meats have been linked to increased risk of colon cancers, whereas breast cancers have been associated with high-fat diets [3,4]. Salted, pickled or smoked foods are linked with an elevated risk of stomach cancers. Low-fiber food and/or high-fat content are associated with colon, prostate, pancreas, breast, endometrium, and ovarian cancers. The clinical management of cancer is based on the type and extent of the disease. Most people undergo a combination of treatments, such as surgery along with chemotherapy and radiation therapy. Various other procedures, such as photodynamic and thermal therapy, immunotherapy, and gene therapy have also emerged as novel treatments for cancer. Phytonutrients are bioactive substances found in plants and are known for their antioxidant and anti-inflammatory effect on humans. Among these phytonutrients, flavonoids and anthraquinones are known to protect the body against various types of cancers [5,6]. Approximately 5000 individual phytochemicals have been identified, and evidence suggests that the additive and synergistic effects of these phytochemicals are responsible for their potent antioxidant and anticancer activities. These phytochemicals play a vital role in apoptosis, cell cycle arrest, the inhibition of angiogenesis, enzyme inhibition, and the modulation of nuclear receptors [7]. This review compiles useful information from all available library databases and electronic searches, including Web of Science, Scopus, Google Scholar, etc., from the period of 1998 to 2021. It highlights the genotoxic and carcinogenic nature of certain food items. It also discusses the traditional and novel treatment modalities of cancer.

## 2. Cancer Mechanism

Cancer is a genetic disease that can arise from a combined effect of multiple external factors along with internal genetic changes. The development of this malicious disease at a cellular level involves somatic mutation in the DNA followed by exposure to carcinogenic factors [6]. This somatic mutation implicates translocation and the strengthening of particular genes; this translates to a distinctive expression of the cell manner named reformed genes. These reformed genes are identified as proto-oncogenes. The genetic damage that occurs mostly becomes irreversible due to numerous cell duplication sequences.

Any agent that prompts mutations or DNA destruction in a cell structure is considered to be genotoxic. A genotoxin can act via direct as well as indirect mechanisms. Ethylene imine and its chloromethyl ether are examples of direct-acting genotoxins. Genotoxins such as hepatitis B virus and aflatoxin are implicated in the etiology of hepatocellular carcinoma, while alcohol and tobacco are risk factors for oral cancer. Indirect-acting genotoxins require metabolic activation to elicit a tumorigenic response. Examples include polycyclic aromatic hydrocarbons and aromatic amines, which are linked to lung cancer and bladder cancer, respectively [8].

It is widely believed that diet and lifestyle strongly impact cancer development. Several carcinogenic and mutagenic constituents are present in our food [9,10]. There is concern over the risk that the pesticide content of commercially grown food items poses. Certain naturally occurring carcinogens, such as pyrrolizidine alkaloids, have been identified in plant products, such as in commonly consumed herbal teas [11,12,13]. Hydrazine in edible mushrooms, and safrole and alkenylbenzene in spices and flavorings have shown carcinogenic properties. In addition, mycotoxins such as aflatoxin present in foods spoiled by fungus have been shown to induce cancer and impair the immune system [14,15].

Oxidative damage is associated with tumor formation. Free radicals which are created by oxidative stress led to DNA damage. These free radicals, if left unrepaired, could cause base mutation, DNA cross-linking, strand breakage, and chromosomal fracture and reorganization [16,17]. Phytochemicals present in our diet have the potential to modulate cancer development and retard tumor growth due to their free radical scavenging capacity. They may positively affect processes of cell signaling, oxidative stress response, and inflammation. There is abundant evidence on the beneficial effects of flavonoids, carotenoids, phenolic acids, and organosulfurs on the downregulation of certain carcinogenic pathways [18,19].

## 3. Types of Cancer and Their Causes

Cancers are classified either according to the kind of tissue from which they originate from, or the organ where they first developed. In addition, some cancers are of mixed types. Development, progression, and incidence of cancer occur at a slow rate, and can take several years to manifest.

The consumption of large amounts of food is one of the key factors in the development of cancer. In developed countries, 30% of all cancer cases have been attributed to poor dietary habits [15]. However, in developing countries, the contribution of diet to cancer risk is relatively less, and accounts for about 20% [16]. It is indicated that poor diet may contribute to 50% of all breast cancers, 70% of colon cancers, and 50% of gallbladder cancer cases [2]. A substantial positive relationship has been established between obesity and high death rates due to various types of cancers such as pancreatic, uterine, kidney, esophageal, breast, and cervix. Researchers believe this is largely due to the inflammation caused by visceral fat [20,21]. Overweight and obesity represent about 20% of all cancer-related deaths in women and 14% in men [22].

**Figure 1:**
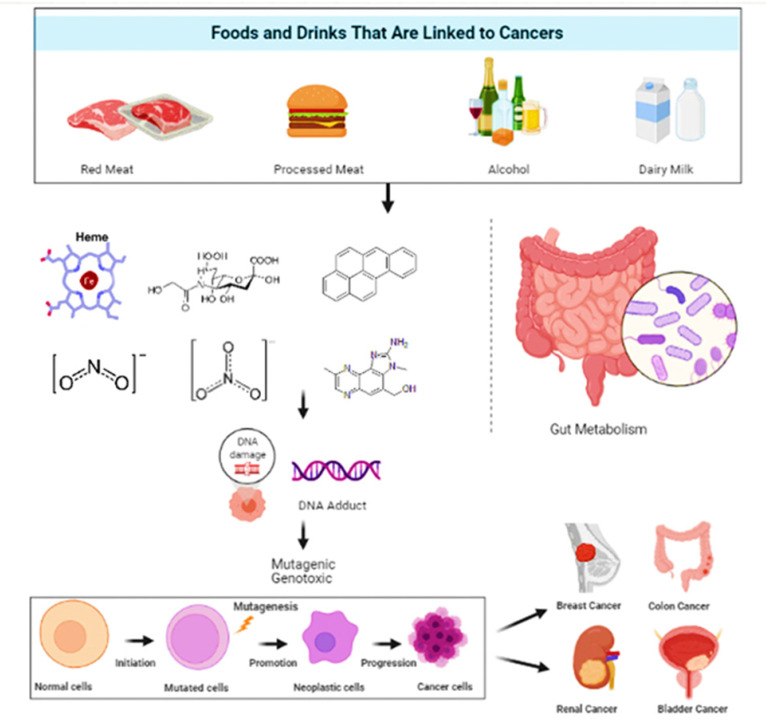
A pictorial representation of the types of food and their effect on selective cancer.

It is estimated that 30–40% of cancers could be avoided by consuming healthy diets, leading a physically active life, and maintaining a healthy body weight [23,24]. Epidemiological research has constantly revealed that a high intake of whole grain products, vegetables and fruits is strongly linked to less deaths due to cancer and cardiovascular ailments, the top two causes of deaths globally [25,26]. Figure 1 gives a general insight on the relationship between food and cancers.

### 3.1. Colon Cancer

Colorectal cancer is the third leading cause of cancer-related death in males, second in females [27], and ranks fourth in cancer-related deaths globally [24,27]. It is observed that there is a higher incidence of colon cancer in developed countries (Oceania and Europe) than developing countries such as Africa and Asia [19,20]. Several studies have revealed that the Western dietary pattern correlates with an elevation of colorectal cancer, whereas diets rich in whole grains and fibers have been linked with reducing colorectal cancer [28,29,30,31,32].

It was also reported in a meta-analysis that a substantial positive correlation existed between the consumption of red meat and colon cancer [33]. On the contrary, white meat consumption does not increase the risk of colon cancer, but the heme content of red meat increases the risk tenfold. Cooked or fried protein-rich foods such as fish and meat are the principal sources of mutagens [34]. Amino acids present in protein tends to react with hexoses and creates hetero aromatic moieties that condense with creatinine, forming imidazo moieties of heterocyclic amines.

In vivo studies to determine the involvement of gut bacteria in mutagenic activation have revealed high mutagenic levels in the urine and feces of rats fed a fried meat diet as compared to germfree rats. A link between a meat rich diet and colon and rectal cancer was hence established [35,36].

A high-fat diet has been suggested to enhance bile acid formation and exert neutral sterols that promote colon carcinogenesis. Dietary fats stimulate fecal bile acid concentration. The primary bile acids include cholic and chenodeoxycholic acid; cholic acids are converted to secondary bile acids (lithocholic and deoxycholic acids, respectively). Secondary bile acids could act as tumor developers, as shown in animal investigations [37,38].

Chronic inflammation has been stated to play a part in developing many forms of cancers, including colon cancer [39]. Carbohydrates, total fat, cholesterol, proteins, saturated fatty acids, and trans fats are pro-inflammatory dietary substances. On the other hand, fibres, minerals, vitamins, isoflavones, polyunsaturated fatty acids, β-carotene and anthocyanidins have been found to exert anti-inflammatory properties [20]. Some studies suggest that Mediterranean diets are protective against colorectal cancer due to the presence of polyphenols from olive oils, through the modulation of several metabolic pathways involved in carcinogenesis [39].

Regular intake of fermented dairy products has been shown to diminish the risk of colon cancer [40,41,42,43]. Lactobacillales (lactic acid bacteria) found in fermented dairy products reduces pro-carcinogen load in the intestine by lowering the concentration of enzymes that convert pro-carcinogens into carcinogens, including β-glucosidase, β-glucuronidase, nitroreductase, and azoreductase [44]. It was also found that occasional curd consumption also had protective effect against colon cancer. Animal trials have been conducted to investigate the effects of probiotics on cancer incidence over a 20-week period; rats fed on a beef-only diet showed a 77% occurrence of colon cancer, whereas an incidence of 40% was noted when they were fed beef along with *Lactobacillus acidophilus* [45].

### 3.2. Breast Cancer

It has been observed that the development of cancer in some sensitive tissues, such as the breast, is probably related to hormonal imbalance [46]. Breast cancer is caused when the DNA in breast cells mutate, disrupting specific functions that control cell growth and division. Risk factors associated with breast cancer include early menstruation/late menopause. Increased levels of endogenous estrogen in postmenopausal women could most likely induce a greater risk of postmenopausal breast cancer. Other risk factors include late age of reproduction, hormonal imbalance, sedentary lifestyle and obesity, and alcohol intake [47,48]. In addition, women who do not breastfeed are at an increased risk of developing breast cancer [49,50,51,52].

Diet may play a role in promoting as well as inhibiting breast cancer development, as concluded by case-control research [53]. The American Cancer Society recommends eating more whole grain foods, vegetables and fruits, and less red and processed meats and sweets. Evidence from several observational studies suggests that a higher intake of omega-3 fatty acids is vital in decreasing breast cancer risk. Fatty acids could influence cancer cell proliferation, angiogenesis, and metastasis [54,55]. Nevertheless, the results of epidemiologic studies concerning dietary factors are inconsistent, and additional research is needed.

A number of epidemiological studies have been conducted to investigate the association between dietary fat intake and risk of breast cancer [51]. Increased consumption of total and saturated fat was found to be positively associated with the development of breast cancer. However, another study conducted on 90,000 nurses found no association between dietary fat consumption and the incidence of breast cancer [39,40,41]. Even though no epidemiological studies provide a strong positive correlation between the consumption of certain types of dietary fat and breast cancer risk, at least a moderate association does seem to exist.

Diets high in glycemic index and glycemic load have been linked to an increased risk of breast cancers. However, a meta-analysis exhibited no causal relationship between postmenopausal breast cancer occurrence and glycemic load consumed [42].

### 3.3. Bladder Cancer

The most common cancer of the urinary tract is bladder cancer, and it is the ninth most common cancer for men [56]. This cancer is substantially more common in males than in females [56]. Smoking is the most important risk factor for bladder cancer, causing about half of all bladder cancers in both men and women. Occupational exposures to aromatic amines used in the dye industry are also linked with bladder cancer. Industries carrying higher risks include leather, rubber, textiles, and paint as well as printing companies. Certain dietary supplements containing aristolochic acid have the potential to cause urothelial cancers, including bladder cancer [57].

No one food by itself can prevent cancer. However, research shows a healthy diet filled with various fruits, vegetables, whole grains and other plant foods could inhibit carcinogenic development, consequently preventing bladder cancer incidence. People who drink a lot of fluids, especially water, each day tend to have lower rates of bladder cancer [58]. Animal studies have shown that the frequency of urination is inversely associated with the level of potential carcinogens in the urothelium. An increase in total fluid intake tends to reduce contact time between carcinogens and urothelium by diluting urinary metabolites and increasing the frequency of voiding. In a randomized experiment, increasing water intake for fifty days by 65 smokers considerably diminished urinary mutagenicity [59].

According to the epidemiological investigations, the relation between total fluid consumption and risk of bladder cancer are conflicted. For example, a health professional’s follow-up study indicated that drinking fluid of 2531 mL per day or above was linked with a 49% decrease in the risk of bladder cancer compared to a lower intake (<1290 mL per day) [60]. On the other hand, a case-control research study performed in the U.S. stated a 41% elevation in the risk of bladder cancer with the intake of total fluid by about 2789 mL/day in comparison to low intake (<1696 mL/day) [61].

Phenolic compounds such as epigallocatechin gallate and resveratrol displayed anticancer action against T24 cells as indicated by in vivo studies [62,63,64,65,66,67,68,69,70]. A study demonstrated that the oligomers of epicatechin, resveratrol and catechin exhibited a noticeable apoptotic influence on the T24 cell line. In contrast, monomers of catechin, resveratrol and epicatechin did not show anticancer influences on T24 cells, since the viability of the cell did not significantly reduce compared to the control sample [71]. However, the antitumor mechanisms of catechin, epicatechin, and resveratrol oligomers are still not clearly understood [58,59]. Certain case-control investigations provide enormous evidence regarding the protective role of carotenoids in bladder cancer, particularly for patients subjected to DNA damage [72,73]. Carotenoids exert their anticancer effect by inhibiting the development of precancerous lesions and scavenging DNA-damaging free radicals.

Data relating dietary habits to bladder cancer survival are limited. The effect of consumption of cruciferous vegetables on bladder cancer survival was investigated, and a strong reverse connection was observed [74]. This result was validated by previous clinical records on isothiocyanates, a group of favorable chemo-protective phytochemicals mostly presented in cruciferous vegetables [75]. High consumption of fats, specifically animal fats, might elevate bladder cancer risk [76,77]. Mutagens associated with bladder cancer etiology may occur during the heating process from fried fatty foods. It is reported that substances such as heterocyclic amines and N-nitroso compounds formed either during cooking or the salt-drying of protein-rich foods could be associated with bladder cancer incidence [78,79,80]. In addition, the intake of persevered meat, such as bacon, ham and sausage, was linked with an elevated risk of bladder cancer [81,82].

Many case-control investigations have stated a considerable elevated risk with the intake of fried eggs [80,81,82,83]. A study showed a strong positive connection with cholesterol intake and it was predicted that half of the estimated cholesterol intake resulted from eggs [84,85]. Nevertheless, the association with the intake of eggs itself was not established. The regular intake of fermented milk containing *Lactobacillus casei* strain Shirota and skimmed milk decreased the occurrence of bladder cancer [86,87,88,89,90,91,92,93,94].

### 3.4. Renal Cancer

Although renal cancer is rare and accounts for around 2% of all cancers, its incidence rate has recently begun to increase worldwide. Little is known about the etiology of this type of cancer; however, smoking, hypertension and obesity are the most established risk factors and are believed to account for almost 40% of all factors, in addition to specific dietary factors [95,96]. A number of studies have attempted to determine the impact of macronutrients and micronutrients on cancer risk [97,98]. Numerous epidemiological investigations have established an inverse relationship between a healthy diet and risk of cancer. Dietary fiber, one of the most commonly used macronutrients, demonstrated many biological activities.

In Europe, a meta-analysis study was conducted to examine the link between renal carcinoma occurrence and the dietary consumption of carbohydrates, protein, fat, fiber, and many other factors. The results showed that there was no correlation between the intakes of macronutrients and renal cancer risk. The results supported the theory that dietary fiber consumption is negatively linked with renal cancer risk. A cohort study conducted in the United States revealed that intake of total fiber was correlated with a significant reduction in renal cancer risk, by about 15–20% [99]. Plant-based and fiber-rich diets high in vegetables and fruits are recommended to prevent cancer and chronic conditions associated with renal cell carcinoma [100]. Since renal cell carcinoma is an obesity-related malignancy, consuming fiber may be protective as it enhances satiety and results in loss of weight by increasing stool bulk and its transit period [101]. Moreover, fiber may also regulate postprandial blood sugar by impeding the entry of glucose into the bloodstream [102]. Butyrate, a short-chain fatty acid produced by dietary fiber fermentation has displayed antineoplastic activity. Additionally, fiber might also decline renal cancer risk by controlling systemic inflammation [103].

A case-control research study has indicated a positive correlation between renal cell carcinoma risk and high energy consumption (protein and fat) mainly from animal foods, and adverse associations with polyunsaturated fat consumption [104,105]. Carbohydrate intake was found to be implicated in several cancers, including renal cancer. An Italian case-control study suggested that foods with high glycemic loads (GL) and glycemic index (GI) were connected with a rising risk of renal cancer [24]. Hypertensive and obese individuals on a high-GI diet are at 2.7 times greater risk of developing renal cancer than individuals without these health issues. It was established in the same study that GI, but not GL, was linked with renal cancer incidence, suggesting that the quality of carbohydrates might perform a more significant role compared to their quantity.

Some foods contain ingredients that can trigger or worsen inflammation, such as processed and sugary foods [106]. Numerous experimental and clinical investigations suggest tumor progression with the up-regulation of pro-inflammatory molecules. Several studies have linked an increase in cancer risk with the inflammatory potential of a diet [79,80,81,82]. For example, the Western diet comprising high-fat dairy products, refined grains, and red meat has been linked with increased levels of pro-inflammatory molecules (interleukin-6, fibrinogen and C-reactive protein) [107]. On the other hand, the Mediterranean diet characterized by high olive oil content, whole grains, green vegetables, and fruits has been correlated with lesser inflammation [108]. A study observed a positive relationship between the inflammatory potential of diet and renal cancer among older women in Iowa [88]. This outcome supports the suggestion that those having pro-inflammatory foods are at a greater risk [109,110,111,112,113,114,115,116,117,118,119,120].

Nevertheless, the indications from potential investigations are scarce, and the World Cancer Research Fund established that there is no reliable information for an association between any nutrients or foods and renal cancer risk [121,122,123]. Prior case-control studies have proposed that a high consumption of animal products is linked with an increase in renal cell carcinoma, even though information from probable data is restricted [124]. A Swedish investigation indicated that a diet rich in fruits and vegetables and modest alcohol intake was associated with increased risk [89]. The findings collected from a population-based case-control investigation of renal cancer performed in the United States supported the protective role of vegetables and considered high meat intake to be deleterious [125]. On the contrary, the European prospective investigation into Cancer and Nutrition observed no correlation with fruit and vegetable consumption [126]. A recent meta-analysis of 13 observational studies proposed a reverse association between vitamin E intake and renal cancer risk [122]. Another study showed an increased risk of renal cancer linked with the consumption of fried/sautéed meat and low intakes of Vitamin E or magnesium. Although varied conclusive results are established about diet and renal cancer, the evidence proposes a possible role of nutrition and renal cancer development. The food groups considered to be protective are green leafy vegetables, fruits and whole grains, nuts, low-fat dairy, and the food groups that showed greatest increase were butter, fried food, and alcohol [121,127].

## 4. Carcinogenic Food Components

Genotoxicity is the property by which chemical agents can damage genetic information within a cell and may induce mutations that lead to cancer. The studies on humans are limited; however, several commonly consumed foods and their components have been screened for their carcinogenic effects on various animal models (Table 1).

**Table 1:** Genotoxic effects of specific foods and site of cancer.

Sites	Food	Carcinogenic Effect/Clinical Studies	Reference
Breast	Red meat	(1) Increasing consumption of red meat was associated with an increased risk of invasive breast cancer. (2) Dietary heme iron, fat, and N-glycolylneuraminic acid are indicated to possibly increase tumour formation, as these compounds are found in red meat.	[119,120]
	Alcohol	(1) Increased circulating levels of estrogen in both premenopausal and postmenopausal women, which might occur through reduced steroid degradation and increased aromatase activity, enhances the transcriptional activity of ER. (2) Contributes to carcinogenesis partly through oxidation from alcohol metabolism and oxidative stress from the production of the alpha-hydroxyethyl radical, a reactive oxygen species, then metabolized to acetaldehyde.	[121,122]
	Dairy Milk	(1) Higher intakes of dairy milk were associated with a greater risk of breast cancer	[123]
Colon	Red meat	(1) High intakes of red and, in particular, processed red meat in unbalanced diets contribute CRC development, PAHs, and HCAs, and dietary NOCs can initiate mutations. (2) A dose–response relationship between heme iron and the promotion of colon carcinogenesis through the fat peroxidation pathway and the N-nitroso pathway where the catalytic role of heme iron from red meat or nitrosyl heme from processed meat is involved in the endogenous production of NOCs, and production of malondialdehyde, which is a carcinogen. Comparatively, heme iron promotes reactive oxygen species (ROS) production, which induces genetic mutations. (3) Participants who reported consuming an average of 76 g/day of red and processed meat compared with 21 g/day had a 20% (95% confidence interval (CI): 4–37) higher risk of colorectal cancer.	[124,125,126]
Bladder	Red meat	(1) Processed meat may be positively associated with bladder cancer risk (red meat was linearly associated with bladder cancer risk in case-control studies, with a pooled RR of 1.51 (95% confidence interval (CI) 1.13, 2.02) for every 100 g increase per day) (2) Intake of processed red meat was significantly associated with the incidence of bladder cancer after multivariate adjustment (highest vs. lowest quintile: HR, 1.47; 95% confidence interval (CI), 1.12–1.93; *p*-trend = 0.008). In contrast, there was only a suggestive but no significant association between the intake of total processed meat and bladder cancer risk after multivariable adjustment (highest vs. lowest quintile: HR, 1.16; 95% CI, 0.89–1.50; *p*-trend = 0.073). (3) Increased BC risk was found for a high intake of organ meat (hazard ratio comparing highest with the lowest tertile: 1.18, 95% CI: 1.03, 1.36, *p*-trend = 0.03) (4) Liver and salami, pastrami or corned beef were found to be associated with increased risk of bladder cancer. Consumption of meats with high nitrate/nitrite, high amine and heme content.	[127,128,129,130]
Renal	Meat	(1) BaP intake, a PAH in barbecued meat, was positively associated with RCC. (2) A meta-analysis indicates a significant positive association between red and processed meat intake and RCC risk (large prospective cohort study observed increased risk of RCC with high consumption of nitrate and nitrite, the precursor of NOCs, and total RCC (hazard ratio = 1.28, 95% CI, 1.10–1.49)	[131,132,133]

Our food selection is influenced by availability and the culture we live in. Moreover, based on our experience, we tend to avoid foods that can cause illness. We ignore many beneficial foods due to lack of knowledge about their nutritional content, taste, faith, societal associations, or cost. There are estimated to be about 250,000 flowering plants, of which 11,000 are used as foods, spices, or flavoring agents, including vegetables, fruits, and nuts. Therefore, the foods we eat are simply a small percentage of those available around us [128]. The major nutrients in our food are carbohydrates, proteins, fats, vitamins and minerals. In addition, fibers and water are also present, which are needed by our body. It is useful to review the functional and beneficial roles of these naturally occurring components, along with their effects on cancer development.

In many countries food has a crucial role in influencing cancer incidence. The knowledge of major sources of macro- and micronutrients is important in order to understand differences in the diet–cancer link in various geographical areas, and to provide better nutritional guidelines. The type and amount of food consumed can have both beneficial and adverse (carcinogenic) effects on the human body [93,94,95,96,97,98,99,100]. As dietary habits are linked to one-third of all cancers, it is critical to look for genotoxic substances or contaminants in foods. Much emphasis has been placed on cooked, uncooked, fermented, and fresh food materials. Several lines of evidence indicate that cooking conditions and dietary habits can contribute to human cancer risk through the ingestion of genotoxic compounds such as acrylamide, heterocyclic amines, and polyaromatic hydrocarbons found in heat-processed foods [101,102,103,104,105,106,107,108,109,110,111,112,113,114,115,116]. The presence of several highly mutagenic substances in cooked meat and fish has been pointed out by many researchers in the past [116,117]. The heating process releases genotoxicants such as aromatic hydrocarbons and heterocyclic amines in beef through processes involving creatin(in)e, sugar, and amino acids [117,118]. Heterocyclic amines are relatively new as dietary genotoxicants; however, they have been found to induce breast, colon, and prostate cancers in animal research. These intoxicants promote carcinogenesis by causing DNA damage and gross chromosomal aberrations. The total caloric intake also has a significant impact on cancer incidence.

### 4.1. Effect of Carbohydrate on Carcinogenesis

Carbohydrates are a broad category of biomolecules. On the basis of few preclinical findings, carbohydrates have been ascribed a deleterious role in the field of cancer research. Carbohydrate intake has been hypothesized to modulate cancer risk depending on the amount and type consumed. Certain studies have reported that carbohydrate consumption induces microbial and epigenetic modulations as well as endocrine and systemic alterations that may influence cancer development [129,130]. Many in vitro and animal trials have presented various mechanisms through which carbohydrates influence cancer development. However, epidemiologic evidence linking dietary carbohydrates to cancer development has remained uncertain [131].

The links between dietary carbohydrates and cancer risk are hypothesized to involve mechanisms that directly implicate players in insulin-mediated pathways across various tissues, as well as through the modulation of IGF-1 bioactivity. One of the primary pathways in cellular proliferation is the insulin/IGF-1 signaling axis which plays a critical role in glucose metabolism.

Insulin interaction with the insulin receptor (IR) is crucial to glucose uptake and energy homeostasis [132]. Animal models have demonstrated that insulin-IR signaling activates signal transduction pathways directly associated with cellular proliferation. Specifically, the hyperstimulation of IR and its interaction with circulating insulin is a hallmark of various cancers [133]. Many observational studies have proven the association between hyperinsulinemia and enhanced risk of adiposity-related cancers [134,135].

Dietary fibers are mainly indigestible complex carbohydrates mainly found in plants. Fibers consist of pectin, lignin, cellulose, and hemicellulose. Mammalian digestive enzymes do not break down fiber, and are moderately metabolized by colonic microflora. Some fibers are water-soluble, and some are insoluble. The consumption of fiber-rich foods, particularly those high in pentoses, is associated with decreased colon cancer risk [136].

A Cochrane systematic review suggested that dietary fiber may reduce the risk of adenomatous polyps of the colon, which are believed to be precursors of colon cancer [137]. Studies have suggested that protective effects of fiber may be associated with lignan found in whole grain foods. Lignans are a group of diphenolic compounds that exert cytostatic activity against colon cancer cell lines [138]. The American Institute of cancer research (AICR) recommends 30 g of dietary fiber each day to lower cancer risk. The AICR report reveals that each 10 g increase in dietary fiber is linked with a 7% decline in risk of colorectal cancer [24]. Fibers may play a role in lowering the risk of other cancers, but the evidence is still limited. The data from animal studies are mixed. Some studies revealed protection, whereas others showed no effect. Therefore, further rigorous studies need to be performed to prove the effect of fiber on cancer prevention.

### 4.2. Effect of Fat on Carcinogenesis

Many case studies have found positive associations between breast cancer and dietary fat intake; however, cohort studies have failed to replicate the same findings. According to several studies, the consumption of red meat is strongly associated with colon cancer due to mutagenic heterocyclic amines which are found in cooked meats [139]. High intake of animal and saturated fats may also be associated with prostate cancer risk [140].

Diets high in fat have augmented the process of carcinogenesis as exhibited in numerous models [141]. The effect depends on the type as well as the amount of fat consumed. Vegetable oils containing polyunsaturated fatty acids of the linoleic acid family (n-6) are known to enhance mammary tumorigenesis, but a fish oil containing polyunsaturated fatty acids of the linolenic acid family (n-3) had an inhibitory effect at higher levels of intake.

At present, the exact mechanism by which dietary fat modulates carcinogenesis has not been elucidated. However, it can be concluded that the influence on synthesis of prostaglandins and leukotrienes may be the universal mechanism by which dietary fats modulate carcinogenesis [142]. Certain studies have reported the role of dietary fat in altering gene expression which could lead to cancer development [143].

### 4.3. Effect of Protein on Carcinogenesis

The significant effect of dietary protein on carcinogenesis appears to be due to its caloric value. Excessive dietary protein increases colonic ammonia levels, and subsequently ammonia may enhance the development of chemically induced colonic tumors [144]. However, limited epidemiologic studies have reported any implication of dietary protein in cancer development. Some studies show associations of colon and breast cancers with animal protein which is considered to be a carcinogen [145].

### 4.4. Effect of Micronutrients on Carcinogenesis

An inadequate diet has detrimental effects on the immune system and various metabolic functions of the body, and it lowers tolerance to cancer treatment. Various epidemiologic and experimental evidence suggests that several micronutrients, including vitamins and minerals, contribute to cancer prevention. Diets lacking these micronutrients could be associated with an increased risk of cancer [146,147,148,149]. Micronutrients such as vitamin C, vitamin E, retinoids, and selenium are not only antioxidants, but also have many essential metabolic functions. They have immunomodulating and apoptosis-inducing properties and regulate cell proliferation and differentiation. In vitro, animal, and human studies have shown that antioxidants reduce cancer cell growth through a variety of mechanisms, including an increase in cell differentiation and apoptosis [150,151,152,153,154].

## 5. Anticancer Bioactive Compounds

Cancer is a devastating disease that has claimed many lives. Natural bioactive agents obtained from plants are gaining popularity for their anticancer activities [155,156,157]. Several studies have found that plant-based bioactive compounds can enhance the efficacy of chemotherapy while also ameliorating some of the side-effects. There is an increasing number of reports which show that many phenolic compounds have potential inhibitory effects on cancer invasion and metastasis. Each medicinal plant has different bioactive compounds that act synergistically to produce the desired protective effect [156]. Natural compounds such as flavonoids, alkaloids, saponins, terpenes, and lignans play an essential role in suppressing cancer cell-activating enzymes, proteins, and signaling pathways [157,158,159,160,161,162,163,164,165,166,167,168]. Various other natural compounds with potent anticancer activity are taxol, camptothecin, vinblastine isolated from Catharanthus roseus, Camptotheca acuminate, and Taxus brevifolia [169,170]. Table 2 lists various anticarcinogenic food items along with their components.

**Table 2:** Anticarcinogenic Food Components.

Sites	Food	Constituent	Anticancer Effect	Reference
Breast	Strawberry	Not mentioned	Induces the intrinsic pathway of apoptosis in breast cancer cells, inhibits tumor progression in mice	[134]
	Pomegranate (*Punica granatum* L.)	Ellagic Acid	Murine breast cancer WA4 cell line inhibited with induction of cell cycle arrest at the G0/G1 phase and apoptosis through caspase-3 activation, reduced cell proliferation and induced apoptosis in MCF-7 cells, antiangiogenic potential (significantly inhibited tumour growth and VEGF receptor 2 (VEGFR-2) phosphorylation), produced synergistic cytotoxic effects	[135,136,137,138]
	Rosemary	Carnosic acid	Decreases cell viability and proliferation, enhances the effect of chemotherapeutics, increases apoptosis and decreases cell transformation.	[139,140,141,142]
	Blueberry	Not mentioned	Tumour volume and multiplicity reduced, down-regulation of CYP1A1 and ER-α gene expression, controlling E2 metabolism	[143,144]
	Saffron	Crocetin	Inhibiting invasiveness	[145]
	Red chilli pepper	Capsaicin	Induces cell death, inhibiting invasion and migration	[146]
	Black seed oil	Thymoquinone	Interferes with PI3K/Akt signalling and promotes G(1) arrest, down-regulates TWIST1 and EMT, inhibits NF-κB; down-regulates p38 MAPK via the generation of ROS; inhibits TWIST1 expression and controls cancer cell metastasis by regulating EMT	[147,148,149]
Colon	Galangal	Galangin	Induces cell death, induces the activation of caspase-3 and caspase-9	[150]
	Black raspberry	Ellagitannins Anthocyanins	Regulates cell cycle and apoptosis of HT-29/HT-116 cell lines mRNA expression of β-catenin and c-Myc, downstream of the Wnt pathway, and cell proliferation decrease; apoptosis increases, DNMT1 decreases	[151,152]
	Pomegranate	Urolithins A and C Urolithin A	Inhibits HT-29 cells proliferation via G0/G1 and G2/M arrest, induction of apoptosis inhibits canonical Wnt signalling pathway	[153,154]
	Rosemary	Carnosic acid	Cell proliferation decreases, cell cycle arrest and apoptosis increase	[141,155,156,157]
	Strawberry	Not mentioned	Nitrotyrosine, phosphorylation of PI3-kinase, Akt, ERK and NF-κB, expression of TNF-α, IL-1β, IL-6, iNOS and COX-2 well as activity of iNOS and COX-2 decrease	[158]
	Onion	Se-Methyl-l-selenocysteine	Induces apoptosis	[159]
	Oregano	Carvacrol	Inhibits proliferation and induces apoptosis	[160]
	Black seed oil	Thymoquinone	Induces apoptosis by up-regulating Bax and inhibiting Bcl-2 and activating caspases -9, -7, and -3 and induction of PARP cleavage; blocks STAT3 signalling via inhibiting JAK2- and Src-mediated phosphorylation of EGFR tyrosine kinase.	[161]
Bladder	Turmeric	Curcumin	β-catenin expression was significantly up-regulated, cell proliferation, migration and invasive ability were reduced.	[162]
	Black seed oil	Thymoquinone (TQ)	Inhibits proliferation and induces apoptosis via endoplasmic reticulum stress-dependent mitochondrial pathway. Attenuates mTOR activity and inhibits PI3K/Akt signalling of T24 cell lines.	[163,164,165]
	Red chilli pepper	Capsaicin	Inhibits tNOX and SIRT1 and thereby reduces proliferation, attenuates migration, and prolongs cell cycle progression.	[166]

Quinoline is a versatile bioactive compound with a wide range of pharmacological activities such as anticancer and anti-inflammatory effects and is regarded to be a superlative molecule in drug discovery. Quinoline scaffold plays an important role in anticancer drug development by inducing apoptosis, cell cycle arrest, angiogenesis inhibition, and nuclear receptor responsiveness modulation [171]. Flavonoids such as catechin, cyanidin, luteolin, epicatechin, quercetin, and kaempferol exert anticancer properties on various cancer cell lines through several mechanisms, such as inhibiting the phosphorylation of epidermal growth factor receptor (EGFR), increasing DNA fragmentation, suppressing signal transduction enzymes, and counteracting angiogenesis. Various alkaloids such as piperine, chabamide, guineensine, piperlongumine, and pellitorine have anti-apoptotic effects. While terpenoids are capable of inducing cell cycle arrest, they down-regulate signal transduction of antiapoptotic protein Bcl-2, and activate pro-apoptotic mediators Bax and Bak [172]. There are 78 flavonoids and xanthones isolated from *Cudrania tricuspidata* which exert inhibitory effects on apoptosis, invasion, and the migration of tumor cells.

The chemopreventive and anticancer activities of Aloe vera are due to bioactive compounds such as anthraquinones, chromones, and polysaccharides [173,174]. They work by inhibiting proliferation, invasion, and the migration of cancer cells. Honokiol, the major bioactive constituent of Magnolia species, exerts its anticancer effect by targeting apoptosis pathways, inhibiting angiogenesis, and regulating cell cycle arrest [175,176].

Osthol is a natural coumarin extracted from Umbelliferae. This bioactive compound inhibits apoptosis via suppressing the activation of different apoptotic proteins, including Smac/DIABLO, poly-ADP ribose polymerase and caspase-3 and caspase-9, as well as suppressing p53. It also can inhibit metastasis through different molecular mechanisms such as suppressing the HGF/c-Met signaling pathway and inhibiting the expression of Smad 2, 3 and 4 [177,178]. Osthol also has a stimulatory effect on the extrinsic apoptotic pathway via increasing the levels of caspase-8 [179]. Many studies have confirmed the efficacy of osthol as a protective and therapeutic agent in various cancers such as cervical, ovarian, colon, prostate, lung, and chronic myeloid leukemia [180].

Emerging evidence supports a link between garlic consumption and decreased cancer incidence due to compounds such as S-allylcysteine and S-allylmercaptocysteine which have antiproliferative potential [181]. Furthermore, some experimental studies have suggested the cytotoxic potential of tanshinone IIA isolated from Scutellaria herbs in breast cancer cell lines [182]. Extracts of Nelumbo nucifera (lotus) were found to suppress cell growth in non-small-cell lung cancer [183,184]. Another bioactive compound isolated from Nelumbo nucifera is 7-hydroxydehydronuciferine. This compound has demonstrated high-quality anticancer bio-functions and has inhibited melanoma tumor growth in vivo and in vitro [185].

Ocimum sanctum, commonly known as Tulsi, is a medicinal herb with immense therapeutic effects. It has various bioactive compounds such as terpineol, caryophyllene, selinene, camphor, decyladehyde, and carvacrol [186]. Carvacrol, is a selective inhibitor of estrogen receptor-alpha, which might be used as a treatment against breast cancer [187]. Pervilleine, isolated from the roots of Erythroxylum pervillei, has shown anticancer effects in combination with vinblastine against multidrug-resistant oral epidermoid cancer cell line (KB-V1) [188].

## 6. Allopathic Cancer Treatment

Effective cancer treatments include surgery, chemotherapy and radiotherapy, as well as newer techniques such as interventional radiology and immunotherapy. The type of treatment that one receives depends on the type of cancer and how advanced it is. Additionally, a combination of treatments is usually needed to achieve the best results [189].

**Figure 2:**
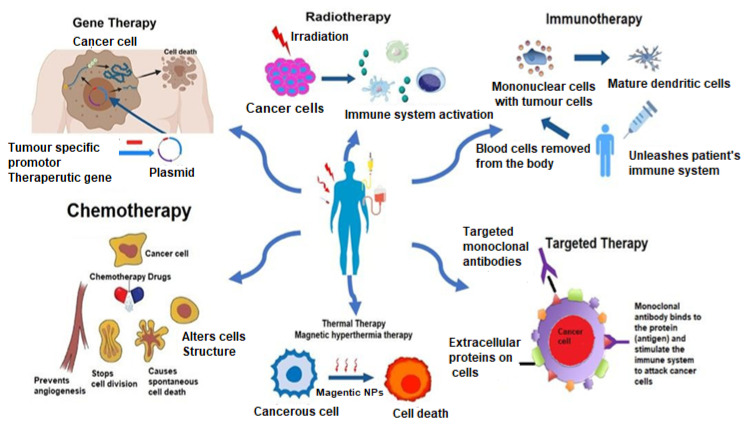
Allopathic treatment involved in cancer therapy.

A brief discussion on the allopathic treatment of cancer is summarized below. Figure 2 illustrates the allopathic treatments involved in cancer. Table 3 enlists the various treatment modalities of cancer.

**Table 3:** List of various treatment modalities for cancer.

No.	Cancer Therapy	Details	Reference
1.	Radiotherapy		[175]
	Radiotherapy individualization based on hypoxia markers	Elevates the oxygen in the blood by breathing in high oxygen levels before and during the irradiation to destroy hypoxic cells using bioreductive compounds or to radiosensitize hypoxic cells using oxygen mimicking drugs.	
	Radiotherapy individualization based on FDG-PET	Fludeoxyglucose (18F-FDG) intensity on a positron emission tomography (PET) image represents the level of glucose uptake by active malignant cells.	
	Markers of DNA repair	One of the best biomarkers for tumor radioresponse of DNA double-strand breaks is gH2AX, a histone protein, which is found after the induction of double-strand breaks.	
	Cancer-stem-cell markers	CD44 is considered as one of the best cancer stem cell markers. A significant correlation of CD44 mRNA expression as well as CD44 immunohistochemical score with local tumor control after radiotherapy was shown in a hypothesis-driven approach.	
	Radiotherapy individualization based on EGFR status	The application of anti-EGFR antibody cetuximab showed locoregional tumor control compared to radiotherapy alone.	
2.	Gene Therapy		[176]
	Oncolytic Virotherapy	It uses replication-competent viruses, which are able to proliferate selectively at tumor cells. It can directly lyse cancer cells, and it also can introduce wild-type p53 tumor suppressor genes into the cells lacking the tumor suppressor gene.	
	Gendicine (Recombinant Human *P53* Adenovirus (Ad5RSV-*P53*))	Gendicine is a non-replicative vector, where the E1 gene is replaced with the p53 cDNA gene. The expression of p53 in tumor cells stimulates the anticancer effect by triggering the apoptotic pathway and inhibiting damaged DNA repair.	
	Oncolytic recombinant ad5 (rAd5-H101)	It was proven to treat refractory nasopharyngeal cancer. Oncorine is an ad5 virus with a deletion in the E1B 55K gene. Host cell p53 gene inactivation is essential for wild-type to block the activation of the apoptotic pathway.	
	Imlygic (Talimogene Laherparepvec)	It was proven that administration of Imlygic causes the apoptosis of cancer cells, improves antigen presentation and increases antitumor response.	
	Rexin-G (Mx-dnG1)	Rexin-G synthesizes cytocidal dnG1 proteins suppress the cell cycle in the G1 phase, leading to the apoptotic pathway of cancer cells.	
3.	Thermotherapy		[177]
	Thermal Ablation Options	It causes destruction and the eradication of the tumor by overheating using temperatures from 55 °C to 100 °C as an external excitation. It can cure many types of cancer such as kidney, liver, lung, rectum, and prostate.	
	Radio Frequency Ablation (RFA)	It uses a high-frequency heating source from 375 to 500 KHz to kill the targeted cells. It has shown a positive result against different kinds of cancers, including breast, liver, and brain.	
	Micro Wave Ablation (MWA)	It uses an electro-magnetic (EM) signal to heat the selected area and stimulate a direct hyperthermic injury. The frequency range begins from 915 MHz to 2.45 GHz.	
	High Intensity Focused Ultra Sound (HIFU)	It sends an ultra sound (US) beam focused on overheating a targeted tissue in order to cause coagulation necrosis. It is highly precise in killing tumors and cures some of the related health issues.	
	LASER Ablation	A LASER (Light Amplification by Stimulated Emission of Radiation) is a monochromatic directed and focused beam of light. It has been used to kill different tumors, especially brain tumors.	
	Cryoablation	Cryotherapy uses a low temperature of −30 to −40 °C to create a freezing zone and generate the destruction of a targeted region. The probe tip is alimented by a source of nitrogen or argon to cool the tissue to −100 °C.	
4.	Chemotherapy		[177]
	Dacarbazine, temozolomide Ethyleneimines: thiotepa, mechlorethamine, chlorambucil, cyclophosphamide streptozocin, carmustine, busulfan	Damage DNA at different phases of the cell cycle. GO phase (resting phase), G1 phase, S phase, G2 phase and M phase. Breast cancer, ovarian cancer, lymphoma, Hodgkin’ disease, multiple, myeloma, sarcoma, lung cancer.	
	Daunorubin, Doxorubixin Epirubicin, Actinomycin-D Bleomycin, Mitomycin-C	Interfere with enzymes involved in DNA replication in all phases of the cell cycle. Leukaemia, breast, cancer, ovarian cancer, intestinal tracts and other various types of cancers.	
	5-fluorouracil (5-FU) 6-mercaptopurine (6-MP) Capecitabine, Cladribine Cloafarabine, Cytarabine Floxuridine, Fludarabine Gemicitabine, Hydroxyurea Methotrexate, Pemetrexed Pentostatin, Thioguanine	Interfere the DNA and RNA formation of cells. Breast cancer, ovarian cancer, intestinal tracts, and other various types of cancers.	
	Topotecan, Irinotecan Etoposide, teniposide	Interfere with topoisomerase such as topoisomerase inhibitor I and II and inhibit the splits of DNA strands during replication. Lung cancer, colon, ovarian, and other gastrointestinal cancers.	
	Paclitaxel, Docetaxel Ixabepilone, vinblastine Estraustine and Vinblastine	Stop cell mitosis or inhibit enzymes associated with protein synthesis required for DNA replication. Breast cancer, lung cancer, lymphomas, colon, other leukaemias.	
	Bortezomib, Carfilzomib, ixazomib	Inhibit the proteasome and the downstream events that lead to selective cell death. Multiple myeloma and mantle cell lymphoma cancers.	
	L-asparaginase	Reduces the level of L asparagine from plasma. As a result, RNA and DNA synthesis are inhibited. Acute lymphocytic leukaemia (ALL).	
5.	Targeted Therapy		[178]
	Monoclonal antibodies EGFR inhibitors: Erlotinib (Tarceva), Afatinib (Gilotrif), Gefitinib (Iressa), Osimertinib (Tagrisso), Dacomitinib (Vizimpro	EGFR inhibitors work by attaching to the EGFR cell surface receptor to block the action of EGF.	
	HER2 inhibitors: Herceptin, Herceptin Hylecta, Margenza, Perjeta	HER2 inhibitors work by attaching to HER2 cell surface receptor to block the action.	
	SMALL MOLECULES Tyrosine Kinase Inhibitors: Imatinib, gefitinib, erlotinib, sorafenib, sunitinib, dasatinib	Tyrosine kinase inhibitors work by blocking the action of receptor tyrosine. Kinases enzymes help to send growth signals in cancer cells.	
	Mammalian target of rapamycin inhibitors (mTOR): everolimus, temsirolimus, sirolimus	mTOR regulates growth factors that stimulate cell growth. The mTOR inhibitors block the activity of mTOR.	
	Poly adenosine diphosphate-ribose polymerase inhibitors (PARP): Olaparib, niraparib, rucaparib, talazoparib.	PARP protein helps to repair damaged DNA in cancer cells. PARP inhibitors act by stopping PARP proteins from repairing DNA in cancer cells.	
	Vascular Endothelial Growth Factor Inhibitor (VEGF): Bevacizumab, Sorafenib, Sunitinib, Nilotinib, Pazopanib, Dasatinib	VEGF forms new blood vessels in cancer cells which helps cell growth. VEGF inhibitors attach to VEGF and inhibit them from growing.	

### 6.1. Chemotherapy

Chemotherapy refers to the use of chemical agents to kill or control cancer cells. These agents induce cytotoxicity frequently, but not exclusively, through apoptosis, a cell-death modality that is non-immunogenic [190]. Many different kinds of chemotherapy drugs are used to treat cancer, either alone or in combination with other drugs or treatments. Paclitaxel is a popular chemotherapeutic agent effective against a broad spectrum of cancers, including head and neck cancer, small-cell and non-small-cell lung cancer, breast and ovarian cancers, colon cancer, melanoma, and multiple myelomas [189].

### 6.2. Radiotherapy

Radiotherapy is used traditionally in combination with surgery or chemotherapy for treating cancer. It is the most important non-surgical modality for the curative treatment of cancer. Radiation–immunotherapy, a combination of radiotherapy and immunotherapy, has also shown effective results in cancer treatment [190,191]. Radiotherapy can cure cancers alone or in conjunction with other treatments; it can also reduce incurable cancer symptoms. A key challenge is to maximize radiation to cancer cells while minimizing injury to the adjacent healthy cells [192]. Technological advancements in the field of radiology, such as intensity-modulated radiotherapy, stereotactic body radiotherapy, and image-guided radiation therapy, have helped maintain a balance between cure and toxicity of treatment. These technologies ensure precise radiation delivery to the target tumor cells and reduce damage to the surrounding healthy cells [193].

### 6.3. Immunotherapy

The immune system plays an important role in regulating tumor growth. Some types of inflammatory responses tend to favor tumor growth, while a tumor-specific adaptive immune response can potentially restrict it [194]. Cancer immunotherapy, also known as immuno-oncology, is a form of cancer treatment that uses a person’s immune system to fight, control, and eliminate cancer. The goal of immunotherapy is to boost or restore the ability of the immune system so that it detects and destroys cancer cells by overcoming the mechanisms by which tumors suppress the immune response [194]. Immunotherapeutic strategies include adoptive cellular immunotherapy, immune checkpoint inhibitors, cytokines, cancer vaccines, oncolytic viruses, and targeted antibodies. The traditional approach to immunotherapy is to increase the frequency of tumor-specific T cells through the administration of cancer vaccines, cytokines, and adoptive cell transfer. Another approach is to trigger innate immune activation and inflammation in the tumor microenvironment with interferons and Toll-like receptor agonists. The most effective strategy to trigger anti-tumor immune responses is to target various checkpoints of immune cell activation, such as programmed cell death protein 1 (PD1), monoclonal antibodies (mAbs), regulatory T-cells, and cytotoxic T lymphocyte-associated protein 4 (CTLA4) [195]. Immune checkpoint inhibitors have proven to be successful, as these agents appear to overcome the mechanisms by which tumors suppress the antitumor immune responses. It was proven that anti-CTLA-4 antibody ipilimumab increases the survival rate of patients with metastatic melanoma for whom conventional therapies have failed [196,197]. Sipuleucel-T is a therapeutic vaccine found to be effective in prostate cancer which has prolonged overall survival [198].

Vascular abnormalities are the hallmark of most solid cancers; they increase proangiogenic factors such as angiopoietin 2 and vascular endothelial growth factors (VEGF). The rational use of drugs that target these factors helps to stimulate immune response and normalize the abnormal vasculature. They convert an immunosuppressive tumor microenvironment to an immune supportive one and trigger the infiltration of immune effector cells into cancer cells. Vascular normalization and immune responses are reciprocally regulated. Therefore, combining immunotherapies and antiangiogenic therapies might enhance the potential of immunotherapy and reduce the risk of immune-related side effects [199].

### 6.4. Targeted Therapy

In targeted therapy, drugs are designed to precisely target cancer cells without affecting the surrounding normal cells. These are classified as small molecules and large molecules. Small-molecule drugs are small enough to enter a cancer cell and work by targeting a specific substance inside the cell and blocking it, thus destroying the cancer cell. For example, imatinib treats chronic myelogenous leukemia and other cancers by blocking tumor-activating signals [200,201]. Large molecules, such as some mAbs, are big in size and cannot fit into a cell. They work by attacking and destroying proteins or enzymes on the surface of the cell and suppress tumor growth by interrupting the interactions between ligands and receptors. Examples include alemtuzumab used in chronic leukemias, trastuzumab used in breast cancers, and cetuximab for lung, head and neck cancers [202,203].

Antibody-targeted therapy: mAbs-based treatment has been established as one of the most successful therapeutic strategies for both hematologic malignancies and solid tumors. mAbs exert their actions through various mechanisms such as antibody-dependent cellular phagocytosis, antibody-dependent cellular cytotoxicity, apoptosis, complement-dependent cytotoxicity, and the blockage of signal transduction. Efforts are being made to maximize the efficacy and minimize the toxicity of these mAbs by loading them with cytotoxic drugs. Such molecules are called antibody-drug conjugates (ADC), which are believed to be tumor-specific. These ADCs combine the specificity and favorable pharmacokinetics of mAbs with the high cytotoxic potential of small-molecule drugs.

Selected examples of targeted cancer therapy are mentioned below based on their mechanism of action [203,204,205,206]. Molecular targeted therapy enables the delivery of anticancer drugs with high-precision targeting. The therapeutic drug used is often a small-molecule drug that targets markers inside the cell or an antibody that attaches to specific targets on the outer surface of cells. Molecular targeted therapeutic agents act on growth factor receptors, cell surface antigens, and signal transduction pathways which regulate cell death, angiogenesis, and metastasis [206]. Agents used in molecular targeted therapy include mAbs, gene therapy, and immunotherapeutic cancer vaccines. VEGF is a crucial stimulus of angiogenesis and blocking it is an effective approach to treat cancer in humans. VEGF receptors (VEGFR) are expressed in different types of leukemias and hematological malignancies. Dovitinib is a potent inhibitor of VEGFRs and has shown efficacy in metastatic melanoma, metastatic RCC, breast cancer, and acute myeloid leukemia. Ligand-targeted therapy ensures selective drug delivery to pathological cells for both therapeutic and diagnostic purposes with the advantage of limited side effects and toxicity. This targeted approach is based on the discovery that there is an overexpression of receptors on pathological cells as compared to normal tissues.

### 6.5. Therapeutic Cancer Vaccines

Therapeutic cancer vaccines are classified as patient-specific and patient-nonspecific vaccines. Patient-specific vaccines are derived from patient’s cancer cells, whereas patient-nonspecific vaccines are derived by activating a general immune response that may have an anticancer effect in some patients [207]. Therapeutic cancer vaccines target specific tumor-associated antigens by stimulating T-cells’ expansion and infiltration, thus resulting in antigen-specific cytotoxicity. Some common proteins are targeted by therapeutic cancer vaccines, including viral proteins (e.g., hepatitis C virus, human papillomavirus), tissue lineage antigens (e.g., tyrosinase, prostatic acid phosphatase), and oncofetal antigens (e.g., carcinoembryonic antigen) [208].

### 6.6. Thermal Therapy

The thermal ablation of cancer involves techniques that utilize heat (hyperthermia) or cold (hypothermia) to kill neoplastic tissues. It has been recorded that cell necrosis develops at temperatures higher than 60 °C or lower than −40 °C. Subsequently, prolonged exposure to temperatures ranging from 41 °C and 55 °C is effective in destroying tumor cells. Thermal therapy can be performed by using five therapeutic strategies, namely cryotherapy (≤508 °C for >10 min), moderate cooling (0–108 °C for 10 min), low-temperature hyperthermia (39–418 °C for times up to 72 h), moderate temperature hyperthermia (42–458 °C for 15–60 min), and high-temperature thermal ablation therapy (>508 °C for >4–6 min). These five therapeutic approaches can impact the tissues through an increase or decrease in oxygenation, blood perfusion, and cellular metabolisms, thus causing protein denaturation, tissue necrosis, and cellular coagulation [209]. Hyperthermia enhances the sensitivity of tumor cells to radiation. It was proven through different clinical trials that hyperthermia combined with radiotherapy and/or chemotherapy significantly reduced the tumor size of many types of cancers, including melanoma, sarcoma, lung, breast, esophagus, brain cancers, etc. It was noted that the combination of hyperthermia with chemotherapy allowed deeper penetration of drugs into tumor tissues, thus enhancing treatment efficacy [210,211,212]. High tumor interstitial fluid pressure (IFP) reduces oxygenation and causes insufficient blood perfusion, and could impede delivery of therapeutics to the tumor site. Systemic heating may stimulate effective thermoregulatory responses, thus reducing tumor IFP and increasing vascular perfusion within cancer tissues.

### 6.7. Gene Therapy

Gene therapy introduces genetic materials such as DNA or RNA into cancerous cells in order to suppress their growth. This can be achieved by replacing the mutated tumor suppressor gene with a normal one, inhibiting tumor angiogenesis and inhibiting the expression of an oncogene [213]. Thus, gene therapy aims to replace, modify, or delete abnormal gene(s) in a targeted cell. Gene delivery systems comprise viral (or bacterial) vectors and non-viral vectors [214]. The most important viral vectors are retroviruses and adenoviruses, whereas non-viral vectors are naked DNA, particle based, and chemical based. Viral vectors are effective in gene delivery and cell transfection, but their application is limited due to their immunogenicity and toxicity. In comparison, non-viral vectors are less toxic, though they require delivery vehicles to invade different types of cells [205].

## 7. Anticancer Foods Show Efficacy in Modulating Cell Proliferation and Improve Overall Survival

Diet and nutrition are important factors in the prevention of various cancers and have a significant impact on disease outcome in patients during and after therapy. Plant-based foods are rich in cancer-beating molecules such as polyphenols, flavonoids, terpenoids, and botanical polysaccharides. Epigallocatechin-3-gallate, gallic acid, gallocatechi-3-gallate, and lupeol are catechins with anticancer properties and are found in green tea [215]. Multiple mechanisms of action have been implicated by which dietary agents contribute to the prevention or treatment of various cancers. Fruits such as black elderberry, guava, and rosemary have chemopreventive and hemotherapeutic potential, and work by targeting key molecular pathways involving NF-κB, cFLIP, FAS, KRAS, PI3K/AKT and WNT/signaling [216]. Phenolics (procyanidin B2 and B1) found in avocado, apple, and bilberry are promising compounds for cancer prevention [215,217]. Vegetables such as broccoli and cabbage are rich in anticancer compounds such as 1H-Indole-3-methanol, indole-beta-carboxylic acid, and 4-methoxy-glucobrassicin. These compounds target molecular networks that control cell division, apoptosis, and angiogenesis.

In summary, the influence of nutrition in cancer metabolism is undoubtedly a topic of widespread interest. Cancers can be avoided by following a nutritious diet, increasing physical activity, and maintaining a healthy body weight. While “prevention” is probably an exaggeration, risk reduction appears to be backed by research. It has been proposed that the cause of most cancers can be explained in part by the shift from a predominantly plant-based diet to a high-fat, high-sugar diet. According to epidemiologic research, changes in lifestyle and dietary variables may play a role in determining the risk of certain cancers. When a cancer diagnosis is made, many people wonder how lifestyle changes will help decrease tumor progression. Calorie restriction is a well-established dietary strategy for cancer prevention and longevity in animal models.

## 8. Conclusions

This review focused on the mechanisms of some types of cancer, as well as the aspects of possible prevention and treatment strategies, by proposing some anticancer foods that show efficacy in modulating cell proliferation and improving survival in these different types of cancers. Research based on the role of nutrition on cancer development is vast, and it is now clear that nutrition plays a major role in cancer development. Diet is just one of the lifestyle factors that influence the risk of developing cancer. Alcohol, tobacco, lack of physical activity, and obesity are among the others. Phytonutrients found in fruits and vegetables exert a synergistic effect to lower cancer risk through various mechanisms including hormone regulation, the downregulation of certain carcinogenic pathways, or the attenuation of inflammation. The perfect recipe seems to be a well-balanced diet high in lean proteins, whole grains, fruits and vegetables, and low-fat dairy, while being low in red meat, sugar, coffee, and alcohol. There is no scientific evidence that a particular type of diet can cure or treat cancer. However, there is ample evidence suggesting that a healthy diet and lifestyle can help reduce the risk of developing certain types of cancers. Further investigations are required to understand inter-individual and geographical variations in diet and their relative contributions to cancer risk.
